# Neighborhood Crime Risk and Racial/Ethnic Differences in Children's Neural Reactivity to Emotional Stimuli

**DOI:** 10.1111/psyp.14759

**Published:** 2025-01-05

**Authors:** Celeste J. Beauvilaire, Brandon E. Gibb

**Affiliations:** ^1^ Binghamton University (SUNY) Binghamton New York USA

**Keywords:** children, EEG, late positive potential (LPP), neighborhood stress, threat

## Abstract

Research has shown that exposure to higher rates of neighborhood disadvantage and contextual threat increases risk for the development of psychopathology in youth, with some evidence that these effects may differ across racial/ethnic groups. Although studies have shown that direct exposure to stress impacts neural responses to threat‐relevant stimuli, less is known about how neighborhood characteristics more generally (e.g., living in neighborhood characterized by high crime risk, whether or not the individual directly experiences any crime) may impact children's neural responses to threat. To address this question, we examined links between census‐derived indices of neighborhood crime and neural reactivity to emotional stimuli in a sample of 100 children (*M*_age_ = 9.64, 54% girls, 65% non‐Hispanic White) and whether these differ for children from minority backgrounds compared to non‐Hispanic White children. Focusing on the late positive potential (LPP) event‐related potential (ERP) component, we examined neural reactivity to threat‐relevant stimuli (fearful faces) as well as nonthreat‐relevant negative (sad faces) and positive (happy faces) stimuli across low, medium, and high intensities (morph levels). We found that levels of neighborhood crime risk were associated with larger LPP amplitudes for high‐intensity fearful, but not happy or sad faces, but only among children from racial/ethnic minority backgrounds. This suggests that levels of crime risk within one's neighborhood may be a more salient stressor for children from minority racial‐ethnic groups than for non‐Hispanic White children.

## Abstract 2

We examined links between census‐derived indices of neighborhood crime risk and event‐related potential (ERP) indices of neural reactivity to emotional stimuli in a community sample of 7‐ to 11‐year‐old children. We found that higher levels of neighborhood crime risk were associated with larger late positive potential (LPP) amplitudes to full intensity threat‐relevant stimuli (fearful faces) but not nonthreat negative (sad faces) or positive (happy faces) stimuli, but only for children from racial/ethnic minority backgrounds and not for non‐Hispanic White children. These results suggest that living in higher crime areas may differentially impact racial/ethnic minority children and point to a potential neural marker of risk in these youth.

Neighborhood context during childhood and adolescence contributes to the development of self and perceptions of the world that are maintained into adulthood (Dupéré, Leventhal, and Vitaro 2012). Children from lower income neighborhoods have been shown to be exposed to more psychosocial stressors than their peers, including greater exposure to community violence (Evans and English 2002). In turn, children and adolescents from neighborhoods with higher levels of crime are at increased risk for psychopathology (Dupéré, Leventhal, and Vitaro 2012; Jorgensen et al. 2023; King, Huang, and Dewan 2022; Lowe et al. 2016; Ramey and Harrington 2019). Although the precise mechanisms by which this risk is conveyed are not clear, a promising candidate is heightened reactivity to threatening stimuli.

Childhood exposure to threat—in the form of negative life events, childhood abuse, or experiences of corporal punishment—is associated with increased neural reactivity to threat‐relevant stimuli (Cuartas et al. 2021; Gollier‐Briant et al. 2016; Puetz et al. 2020; White et al. 2019). Further, adolescents who have experienced or witnessed violence (e.g., gun/knife violence and physical altercations) exhibit heightened amygdala responses to angry facial stimuli (White et al. 2019). Each of these studies focused on direct experiences and less is known about how contextual markers, such as simply living in an area with higher crime rates, may impact neural reactivity to threat. This said, there is clear evidence from laboratory‐based studies that contextual manipulations of threat, including lack of control over potential threat, increase neural reactivity to both threat‐relevant as well as potentially neutral stimuli as indexed by the late positive potential (LPP) event‐related potential (ERP) component (Bauer, Thomas, and MacNamara 2021; Foti and Hajcak 2008; Imburgio and MacNamara 2019; Klein et al. 2015; MacNamara, Foti, and Hajcak 2009; Qiao et al. 2018; Stolz, Endres, and Mueller 2019). Understanding the potential impact of contextual neighborhood influences is important because many children live in high crime areas, even if they are not personally the victims of crime themselves.

The primary goal of this study, therefore, was to examine links between neighborhood indices of crime risk and children's neural reactivity to threat‐relevant stimuli. We focused on children (aged 7–11 years old) because our goal was to identify potential mechanisms of risk prior to the significant increase in psychopathology that occurs during adolescence (Costello, Copeland, and Angold 2011). Building from earlier research examining contextual threat, we focused on the LPP, which is commonly used to study neural reactivity to emotional stimuli, with larger LPPs observed for emotional or personally salient stimuli than for neutral stimuli (Jorgensen et al. 2023). To determine whether the findings were specific threat‐relevant stimuli (fearful faces), we also included nonthreat‐relevant negative stimuli (sad faces) and positive stimuli (happy faces). We predicted that children from areas with higher levels of neighborhood crime would exhibit larger LPP responses specifically to threat‐relevant stimuli. Given evidence that threat exposure may also increase children's sensitivity to milder or ambiguous displays of threat (e.g., Dieterich, Endrass, and Kathmann 2016; Qiao et al. 2018; Sandre et al. 2018), we examined three levels of emotional intensity (low, medium, and high) for each of the stimuli presented. Although our primary hypotheses focused on high‐intensity threat images, we also anticipated that higher levels of crime risk would be associated with increased LPP responses to the medium morph images.

A secondary aim was to determine whether links between neighborhood crime risk and children's neural reactivity to emotional stimuli may differ for children from minority racial/ethnic backgrounds compared to non‐Hispanic White children. There is evidence for greater neural reactivity to threat‐relevant stimuli in Black compared to White individuals, particularly among those exposed to higher levels of stress (Andrews III et al. 2019; Fani et al. 2021; Harnett et al. 2019). There is also evidence that higher parent‐reported levels of neighborhood disadvantage are associated with greater salience of threat cues in racial/ethnic minority youth than in White youth (Jorgensen et al. 2023). Given this, we predicted that the link between neighborhood crime risk and LPP reactivity to threat‐relevant stimuli would be stronger among racial/ethnic minorities, compared to Non‐Hispanic White children. Finally, to ensure that our findings were not due simply to elevated levels of psychopathology (internalizing or externalizing symp) in children from higher crime areas or differences in family income, we conducted sensitivity analyses to determine whether any observed relations were at least partially independent of these influences.

## 1 Method

### 1.1 Participants

Participants in the study were 100 children and their parents recruited from the community. Because our goal was to recruit a representative community sample with minimal inclusion/exclusion criteria, the only inclusion criterion was that the child be between 7 and 11 years old and the only exclusion criteria was the presence of a learning or developmental disorder, per parent report, that would make it difficult for them to complete the study. The average age was 9.64 (SD = 1.47) and 54% of the children were girls. In terms of race/ethnicity, 65% were non‐Hispanic White and 35% were racial/ethnic minorities (race: 12% African American, 2% Asian, 11% Biracial; ethnicity: 16% Hispanic). The median annual family income based on parent reports was $50,000–$55,000.

### 1.2 Measures

#### 1.2.1 Neighborhood Crime Exposure

Participants' neighborhoods were determined based on the zip code of their current address at the time of study enrollment. Neighborhood crime exposure indices were obtained from CrimeRisk (Applied Geographic Solutions 2015), which is a database containing geocoded information about crime risk indices for multiple types of crime including property (i.e., burglary, larceny, motor vehicle theft) and personal (i.e., murder, rape, robbery, assault) crime rates for each zip code within the target county. The crime risk index, reflecting the relative risk of a crime occurring in an area compared to the national average, was calculated in CrimeRisk from a thorough analysis of crime reports in the target county across a 7‐year period. A score of “100” reflects the national average for total crime risk. In the current study, crime risk scores ranged 13 to 143 (*M* = 76.47, *SD* = 33.95).

#### 1.2.2 Morphed Faces Task

Participants completed a morphed faces task (Burkhouse, Siegle, and Gibb 2014) in which they viewed grayscale faces from a standardized stimulus set of child actors (Egger et al. 2011) displaying a variety of emotions (fear, happy, or sad). Each face was 26.5 cm tall (16° visual angle) and 16.5 cm wide (10° visual angle). The stimuli consisted of emotional and neutral photographs from each actor morphed to form a continuum of 10% increments between the two photographs. Each emotion was represented by four continua (2 male and 2 female actors) for a total of 12 continua. A total of 11 morphed images were used from each continuum, representing 10% increments of the two emotions ranging from 100% neutral to 100% target emotion (e.g., 100% neutral, 0% fear; 90% neutral, 10% fear; 80% neutral, 20% fear). The pictures were presented one at a time in the middle of the screen for 3 s, after which they disappeared and participants were asked to indicate which emotion was being presented using the following four response options for each image: fear, happy, sad, or calm/relaxed. The inter‐trial interval varied randomly between 500 and 750 ms. The stimuli were presented in semi‐random order with the condition that no two images from the same actor were presented consecutively. Each of the 132 images was presented twice for a total of 264 trials, with a rest after every 55 trials. Consistent with previous research (Burkhouse, Siegle, and Gibb 2014; Burkhouse et al. 2019; Jenness et al. 2015), and to provide an adequate number of trials within each morph level, images were binned into three separate morph conditions for analyses: low (10%, 20%, and 30% target emotion), medium (40%, 50%, 60%, and 70% target emotion), and high (80%, 90%, and 100% target emotion).

During the morphed faces task, continuous electroencephalography (EEG) was recorded using a custom cap and the BioSemi ActiveTwoBio system (Amsterdam, Netherlands). The EEG was digitized at 24‐bit resolution with a sampling rate of 512 Hz. Recordings were taken from 34 scalp electrodes based on the 10/20 system. Offline analysis was performed using the MATLAB extension EEGLAB (Delorme and Makeig 2004) and the EEGLAB plug‐in ERPLAB (Lopez‐Calderon and Luck 2014). All data were re‐referenced to the average of the left and right mastoid electrodes and bandpass‐filtered with cutoffs of 0.1 and 30 Hz. EEG data were processed using both artifact rejection and correction. Large and stereotypical ocular components were identified and removed using independent component analysis (ICA) scalp maps (Jung et al. 2001). Artifact detection and rejection were then conducted on epoched uncorrected data to identify and remove trials containing blinks and large eye movements at the time of stimulus presentation. Epochs with large artifacts (> 100 μV) were excluded from the analysis. Consistent with previous studies measuring LPP responses in children (Dennis and Hajcak 2009; Kujawa, Klein, and Hajcak 2012) the LPP was calculated as the mean activity 400–1000 ms following face onset averaged across occipital (O1, O2, and Oz) and parietal (P3, P4, PO3, PO4, and Pz) electrode sites separately for each emotion (fearful, happy, sad) and morph level (low, medium, high).

#### 1.2.3 Internalizing and Externalizing Symptoms

As part of our sensitivity analyses, we sought to determine whether any significant relations were at least partially independent of children's current internalizing and externalizing symptoms. To assess these symptoms, we used parents' reports on the Child Behavior Checklist for ages 6–18 (CBCL; Achenbach and Rescorla 2001). Numerous studies have supported the reliability and validity of the CBCL (see Achenbach and Rescorla 2001). In the current study, both the internalizing (*α* = 0.83) and externalizing (*α* = 0.91) subscales of the CBCL exhibited good internal consistency.

### 1.3 Procedure

Participants were recruited from the community through various forms of advertisements (e.g., television, bus ads, flyers, and newspapers). Upon arrival at the laboratory, parents provided consent and children provided assent. Following this, children completed the morphed face task and parents completed questionnaires including a report of their current address. All study procedures were approved by the University's Institutional Review Board.

### 1.4 Analysis Plan

Hypotheses were tested using SPSS (version 29). Preliminary analyses tested for potential racial/ethnic group differences in neighborhood crime risk. To test our primary hypotheses, we used a repeated measures general linear model (GLM) with level of neighborhood crime risk (treated as continuous) and racial/ethnic group (non‐Hispanic White vs. racial/ethnic minority) included as between‐subjects factors and emotion (fearful, sad, happy) and morph level (low, medium, high) included as within‐subjects factors. We predicted that higher levels of neighborhood crime risk would be associated with increased LPP responses specifically to threat‐relevant stimuli (fearful faces) and that this relation would be stronger for racial/ethnic minority children than for non‐Hispanic White children. Although our hypotheses focused on high‐intensity images, potential interactions with morph level were explored to determine whether similar effects may be observed at medium or low morph levels. All significant interactions were examined to determine the pattern of the interactions.

## 2 Results

Correlations between variables can be found in Table 1. Preliminary analyses revealed that children from racial/ethnic minority groups lived in areas with greater crime risk (*M* = 92.87, *SD* = 28.39) than their non‐Hispanic White peers (*M* = 67.64, *SD* = 33.59), *t*(98) = 3.77, *p* < 0.001, Cohen's *d* = 0.79. This said, there was a clear range of crime risk for minority children (58–143) and non‐Hispanic White children (13–143).

**TABLE 1:** Descriptive statistics and correlations for study variables.

	1	2	3	4	5	6	7	8	9	10	Mean (SD)
1. Racial/ethnic group	—										—
2. Crime risk	−0.36	—									76.47 (33.95)
3. LPP: Afraid/Low	0.08	−0.13	—								11.23 (8.75)
4. LPP: Afraid/Medium	0.13	0.02	0.61	—							12.74 (8.88)
5. LPP: Afraid/High	0.19	0.09	0.51	0.55	—						13.56 (8.54)
6. LPP: Happy/Low	0.23	−0.04	0.59	0.54	0.56	—					11.70 (8.65)
7. LPP: Happy/Medium	0.32	−0.13	0.45	0.53	0.49	0.61	—				10.21 (8.00)
8. LPP: Happy/High	0.21	−0.15	0.52	0.55	0.37	0.57	0.49	—			11.20 (7.81)
9. LPP: Sad/Low	0.14	−0.05	0.61	0.67	0.55	0.68	0.49	0.52	—		11.10 (7.26)
10. LPP: Sad/Medium	0.13	−0.11	0.52	0.59	0.54	0.52	0.40	0.40	0.67	—	12.10 (7.82)
11. LPP: Sad/High	0.12	0.02	0.39	0.50	0.59	0.47	0.54	0.31	0.49	0.47	13.81 (8.63)

*Note:* Racial/ethnic group coded as 1 = Non‐Hispanic White, 0 = Racial/ethnic minority. LPP = Late Positive Potential. Correlations greater than | 0.20 | significant at *p* < 0.05, greater than | 0.26 | significant at *p* < 0.01, and greater than | 0.33 | significant at *p* < 0.001.

To test our hypotheses regarding the link between neighborhood crime and children's LPP responses to emotional stimuli, and how this may be moderated by children's race/ethnicity, we used a repeated measures general linear model with level of neighborhood crime risk (treated as a continuous variable) and racial‐ethnic group (non‐Hispanic White vs. racial‐ethnic minority) as between‐subjects factors and emotion (fearful, sad, happy) and morph level (low, medium, high) as within‐subjects factors. In this analysis, there were significant crime × emotion × morph, *F*(4, 384) = 5.03, *p* < 0.001, *η*_p_^2^ = 0.05, and racial/ethnic group × emotion × morph, *F*(4, 384) = 4.66, *p* = 0.001, *η*_p_^2^ = 0.05, interactions. Importantly, the crime × racial/ethnic group × emotion × morph interaction was also significant, *F*(4, 384) = 3.70, *p* = 0.006, *η*_p_^2^ = 0.04.

**FIGURE 1:**
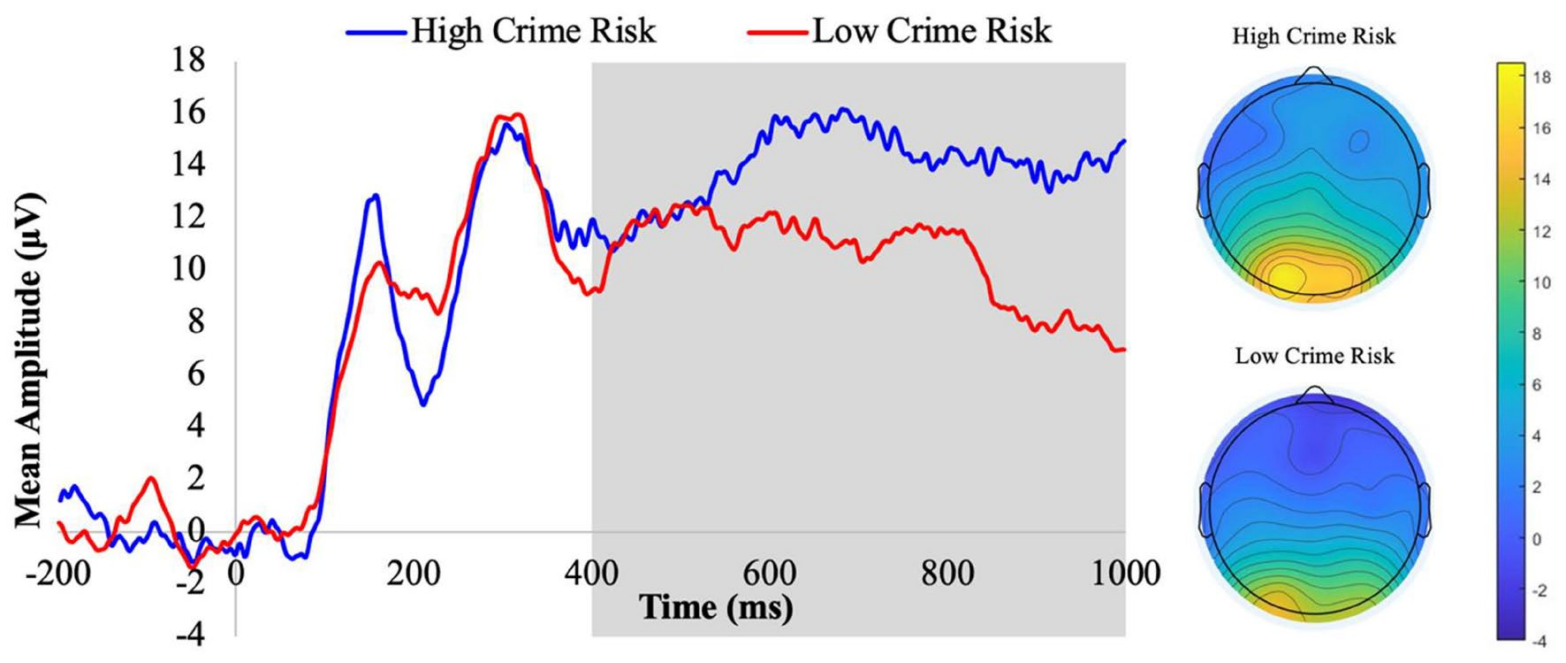
Waveforms and scalp topographies depicting the late positive potential 400–1000 ms following the onset of high‐morph afraid faces in racial/ethnic minority children from high versus low crime risk neighborhoods.

To determine the form of this interaction, we examined the crime risk × emotion × morph interaction separately in minority and non‐Hispanic White children. In these analyses, the crime risk × emotion × morph interaction was significant for racial/ethnic minority children, *F*(4, 132) = 4.89, *p* = 0.001, *η*_p_^2^ = 0.13, but not for non‐Hispanic White children, *F*(4, 252) = 1.36, *p* = 0.25, *η*_p_^2^ = 0.02. Examining this further in children from racial/ethnic minority groups, the crime risk × morph interaction was significant for fearful faces, *F*(2, 66) = 6.99, *p* = 0.002, *η*_p_^2^ = 0.18, but not for happy, *F*(2, 66) = 2.66, *p* = 0.08, *η*_p_^2^ = 0.08, or sad, F(2, 66) = 0.73, *p* = 0.48, *η*_p_^2^ = 0.02, faces. As a final step, we examined the correlation between neighborhood crime risk and LPP amplitude to fearful faces at each morph level in racial/ethnic minority children. Higher levels of neighborhood crime risk were associated with larger LPP amplitudes for fearful faces at high, *r* = 0.36, *p* = 0.03, but not medium, *r* = −0.14, *p* = 0.42, or low, *r* = −0.26, *p* = 0.13, morph levels. Although all analyses were based on continuous levels of neighborhood crime risk, we used a median split to provide a visual depiction of these results (see Figure 1).

To evaluate the robustness of these results, we conducted follow‐up partial correlation analyses to determine whether the relation between higher levels of neighborhood crime risk and minority children's LPP responses to high morph fearful faces was maintained after statistically controlling for the influence of children's current internalizing and externalizing symptoms, as well as children's family income and age. In all cases, the significant relation was maintained (all *p*s < 0.05).

## 3 Discussion

The goal of this study was to examine the link between a census‐derived index of neighborhood crime risk and children's neural reactivity to threat‐relevant stimuli, and whether this link may be stronger among children from racial/ethnic minority backgrounds. We found that higher levels of neighborhood crime risk were associated with larger LPP amplitudes to threat‐relevant images, but only among racial/ethnic minority children and not among non‐Hispanic White children. This finding was specific to full‐intensity threat images and was not observed at lower intensities. It was also specific to threat‐relevant stimuli (fearful faces) and was not observed for nonthreat negative (sad faces) or positive (happy faces) stimuli. Therefore, in addition to living in areas characterized by higher crime risk, children from racial/ethnic minority groups also exhibited greater neural reactivity to threat‐relevant stimuli in the context of higher crime risk. Because the LPP reflects salience of, and sustained attention toward, emotional stimuli, this suggests that levels of neighborhood crime risk may be a more salient stressor for racial/ethnic minority children than for non‐Hispanic White children, which could impact their reactivity to threat‐relevant cues (cf. Jorgensen et al. 2023). Importantly, the relation between neighborhood crime risk and children's LPP reactivity to threat‐relevant images was maintained when we statistically controlled for the influence of children's family income and current symptoms of internalizing and externalizing disorders, suggesting that the relation is at least partially independent of these other factors.

Based on previous research suggesting that LPP amplitudes may be enhanced in the context of ambiguous or uncertain threats (e.g., Dieterich, Endrass, and Kathmann 2016; Qiao et al. 2018; Sandre et al. 2018), we had expected that higher levels of crime risk would be also associated with larger LPP amplitudes for fearful faces at medium morph intensities. However, the relation was specific to high‐intensity stimuli. Sandre et al. (2018) also used a morphed faces paradigm and found that undergraduates reporting a history of at least low levels of childhood abuse exhibited a linear increase in LPP amplitudes with increasing levels of fear intensity, whereas those with no history of childhood abuse did not. However, even in this study, the two groups only differed for full‐intensity fearful faces. It is possible, therefore, that effects would be stronger in the context of uncertain threat (e.g., Dieterich, Endrass, and Kathmann 2016; Qiao et al. 2018) rather than simply for milder levels of a threat‐relevant stimulus. Future research is needed to examine this possibility.

The current results build upon previous research showing that direct experiences with crime and violence are associated with greater neural reactivity to threat‐relevant stimuli (e.g., Cuartas et al. 2021; Gollier‐Briant et al. 2016; Puetz et al. 2020; White et al. 2019) to show that similar patterns of heightened neural reactivity are observed as a function of simply living in an area characterized by higher risk of crime. The current findings also build on previous research showing that risk differs across racial/ethnic groups such that racial/ethnic minorities residing in areas experiencing higher crime rates are at particularly high risk for developing psychopathology (Andrews III et al. 2019) by showing that the link between neighborhood crime risk and neural reactivity to threat‐relevant stimuli is stronger for racial/ethnic minority children than for non‐Hispanic White children. These results suggest that increased neural reactivity to threat may be one mechanism by which living in an area characterized by higher risk of crime increases the risk for psychopathology among racial/ethnic minority children. However, longitudinal studies are needed to more formally test this mediational hypothesis.

Another important area of future research is understanding the mechanisms underlying the stronger link between neighborhood crime risk and neural reactivity to threat in racial/ethnic minority compared to non‐Hispanic White children. Although, historically, people from minoritized backgrounds have been more likely to live in poorer neighborhoods that experience higher rates of crime (Anderson et al. 2003; Ellen and O'Regan 2009), there is also evidence that neighborhood crime is more salient for minority youth. For example, children from racial/ethnic minority backgrounds may have a greater fear of being a victim themselves (McIntyre and Spatz Widom 2011) or may be more reactive to the correlates of living in a higher crime area, such as increased police presence, given that racial minorities have been shown to be differentially impacted by policing (Brunson and Miller 2006; DeVylder et al. 2017; Schleiden et al. 2020). Additional research is needed to examine these and other possibilities. Finally, additional research is needed to examine the degree to which children are aware of the specific forms of crime happening in their neighborhood and if that awareness puts them at further risk.

In summary, this study supports the link between neighborhood crime risk and children's increased neural reactivity to threat‐relevant stimuli, but only among racial/ethnic minority children and not non‐Hispanic White children. Although not examined in this study, this increased neural reactivity to threat may be one mechanism of risk for later psychopathology in these children. Future research is needed to identify potential sensitivity periods for the impact of neighborhood characteristics as well as the impact of moving out of higher crime areas. Research is also needed to better understand mediators and moderators of the link between neighborhood crime risk and minority children's neural reactivity to threat, which may highlight additional targets of intervention to reduce risk for later psychopathology in these children.

## Author Contributions

**Celeste J. Beauvilaire:** writing – original draft, writing – review and editing. **Brandon E. Gibb:** methodology, resources, supervision, writing – review and editing.

## Conflicts of Interest

The authors declare no conflicts of interest.

## Data Availability

The data that support the findings of this study are available from the corresponding author upon reasonable request.
